# Clinical teachers’ professional identity formation: an exploratory study using the 4S transition framework

**DOI:** 10.5116/ijme.61dd.7764

**Published:** 2022-01-28

**Authors:** AASA Santhi Sueningrum, Marcellus Simadibrata, Diantha Soemantri

**Affiliations:** 1 Faculty of Medicine and Health Sciences Universitas Warmadewa, Bali, Indonesia; 2 Department of Internal Medicine, Faculty of Medicine Universitas Indonesia, Cipto Mangunkusumo General Hospital, Jakarta, Indonesia; 3 Department of Medical Education, Faculty of Medicine, Universitas Indonesia, Jakarta, Indonesia

**Keywords:** Clinical teacher, professional identity, 4S framework, transition

## Abstract

**Objectives:**

The aim of this study was to explore factors that may influence the formation of professional identity in clinical teachers, specifically during the transition period from practitioner to teacher.

**Methods:**

This was a descriptive qualitative study. We used Schlossberg’s 4S framework to explore influential factors comprised of the following: situation, self, support, and strategies. This study was conducted in teaching hospitals of a relatively new private medical school in Bali, a province in Indonesia. The participants were 30 clinical teachers who were selected using a maximum variation sampling strategy based on length of work experience, gender, specific educational roles as coordinators, and clinical specialty. Data were derived from three focus-group discussions and 13 in-depth interviews. A thematic analysis method was used to analyse the data.

**Results:**

The thematic analysis revealed that 12 subthemes related to the 4S framework influenced the development of clinical teachers’ identity. It was also shown that reflective ability and community of practice, which was included in the self and strategy factors, respectively, were the two most important factors during the transition period in the development of professional identity.

**Conclusions:**

Factors, both within and outside the self, can either support or hinder the formation of professional identity in clinical teachers. We suggest that when faculty development programs are designed, these factors should be incorporated, such as including a community of practice as part of the formal faculty development programs and the development of a teaching portfolio that nurtures reflective practice.

## Introduction

Clinical clerkship is an important foundational training component in medical school.^1^^-^^3^ Thus, clinical teachers play an important role in the educational quality of clinical rotations because the development of a student’s professional identity occurs during this period. A student’s professional transformation requires that clinical teachers are not only supervisors but also models of the values and professional attributes of the profession.^2^^,^^4^^,^^5^

To successfully perform their roles, clinical teachers require support in improving their professionalism as educators, as most have either minimal or no prior knowledge of educational practices, including appropriate learning activities, curriculum development, and assessments.^2^^,^^6^^-^^8^ Therefore, an understanding of clinical teachers’ views and concepts of their role is needed to support the development of their professional identity as clinical teachers.^9^^,^^10^

Professional identity plays an important role in a teacher’s capacity as an educator in any field, and medical education is no exception. In the process of developing a professional identity, teachers will typically integrate and absorb the knowledge, skills, values, and attitudes of their chosen profession. Educators who are able to successfully integrate the role of teacher into their identities are considered to have formed an emotional attachment to the profession. Accepting one’s professional identity as a teacher is also crucial in maintaining a teacher’s well-being.^11^^-^^14^

Globally, the development of medical education is accompanied by an increasing number of new medical schools. Consequently, more students are completing their education as medical doctors.^15^^-^^18^ Due to the increasing number of medical students, there is an increased need for clinical training programs. New medical schools face many challenges, including the capacity of the health system to accommodate the clinical clerkship phase of medical education programs. Thus, concerns about the quantity and quality of clinical training programs, including that of clinical teachers, have grown.^18^^-^^20^

The increasing number of new medical schools has resulted in a situation in which many clinicians will be obliged to undergo a change in their role from that of a clinician mostly involved in patient care to someone who also teaches medical students during their clinical clerkships. This role change can be challenging, considering that some clinicians did not expect to serve in a teaching capacity. In such cases, clinicians may experience confusion and a lack of preparation in taking on a teaching role. In addition, similar to established medical schools, new medical schools face challenges with retaining faculty.^21^

Each individual has his or her own ability to cope with change, and this ability can be explored by using the 4S framework. Schlossberg postulated that four factors have the potential to influence an individual’s ability to cope with change: (1) the situation factor relates to the characteristics of the change process (e.g., trigger of transition);  (2) the self-factor relates to the personal characteristics that might influence an individual’s perception toward change (e.g., self-efficacy, motivation); (3) the support factor plays an important role in ensuring an individual’s well-being through the change process (e.g., social support); and (4) the strategies factor includes strategies to prevent or mitigate any stresses that might occur during the change process (e.g., coping strategy).^22^^,^^23^

Browne and colleagues used the 4S framework to explore potential factors that contribute to the success of senior medical educators transitioning from clinician to teacher who self-identify as medical educators.^24^ In their study, Browne and colleagues identified the following key factors of a successful transition: (1) ability to associate personal value with the profession of a medical educator as a self-factor; (2) self-control in being involved in medical education as a situation factor; (3) personal support in undergoing role changes as a support factor; and (4) creative strategies and actively seeking information, networking, self-development, and recognition in medical education as a strategies factor.^24^

The development of a professional identity is a continuous process that ideally should foster an individual’s personal and professional growth. It has been observed that the development of a professional identity strengthens an individual’s commitment to their profession.^12^ Therefore, understanding the factors that influence clinical teachers during the transition process will help support the change in a teacher’s role as well as improve faculty retention by enhancing the development of professional identity. The aim of this study was to explore the factors influencing the formation of professional identity in clinical teachers. Furthermore, a better understanding of the experience of clinical teachers in developing their professional identities can provide a basis for faculty development that is both knowledge- and skills-based.

## Methods

### Study design and participants

A descriptive qualitative design was employed to explore the experiences of clinical teachers as they developed their new professional identity and learned to integrate the new identity into their existing professional identity as clinicians.

The participants of this study included 30 clinical teachers who were still actively involved in teaching. Recruitment of the participants was conducted using maximum variety sampling by considering any characteristics that could affect the perspective of the phenomena under study. These included the following: length of experience as a clinical teacher, gender, specific educational roles as coordinators, and clinical specialty (Table 1). The participants were selected from diverse backgrounds to provide a range of viewpoints and an in-depth understanding of the formation of professional identity in clinical teachers.^25^

**Table 1:** Participants’ characteristics

Variables	Number
Gender
Male (M)	14
Female (F)	16
Length of experience as clinical teacher
< 3 years	19
> 3 years	11
Specific educational roles as coordinators
Yes (C)	12
No (NC)	18
Clinical specialty	
Surgery (S)	5
Pediatrics (Pd)	2
Radiology (R)	2
Neurology (N)	3
Psychiatry (Py)	1
Ophthalmology (Op)	3
Anesthesiology (A)	2
Internal medicine (I)	4
Obstetrics and gynecology (Og)	3
Dermatology and venereology (D)	3
Otolaryngology-head and neck surgery (Ot)	2

Ethical approval was obtained from the Ethics Committee of the Faculty of Medicine and Health Sciences Universitas Warmadewa in Bali, Indonesia. We also obtained permission from both teaching hospitals. Participation in the study was voluntary, and all data were presented anonymously. All participants provided their consent prior to any data collection.

### Interviews

Guided interviews consisted of 17 questions that were developed based on a literature review of the 4S framework and professional identity development. The questions were created to explore the factors related to situation, self, support, and strategies that were likely to influence the participants’ transition process in becoming clinical teachers. We prepared questions to explore each factor, such as questions regarding the motivation for and the impact of becoming a clinical teacher to explore the self-factor. The situation factor was explored using questions pertaining to perceptions about the transition process. The support factor was explored using questions related to the support they perceived to be important or helpful while undergoing the transition process. Finally, for the strategies factor, we asked participants which strategies they used as coping mechanisms throughout the transition process.

### Data collection methods

Three focus groups (FGs) and 13 in-depth interviews were conducted to collect data from the participants from March to April 2020. Seventeen participants were distributed into three focus groups for each hospital: (1) clinical teachers with experience >3 years; (2) clinical teachers with experience <3 years; and (3) clinical teachers who held the position of leader/coordinator. The number of participants in each focus group was between five and six. The other 13 participants were individually interviewed. The researchers acted as moderators and guided the FGs/interviews, and asked open-ended questions based on the interview guidelines. The FGs/interviews were recorded with a voice recorder. Afterwards, all recordings were transcribed verbatim.

### Study setting

Similar to other countries, there has been an increasing number of new medical schools in Indonesia, with an even larger increase following a regulation in 2010 that mandated that every island in Indonesia have at least one medical school.^26^ Currently, there are 89 medical schools in Indonesia. Since 2010, 19 new medical schools have been opened.^27^ This study was conducted at both the main teaching hospital and a satellite teaching hospital of a relatively new private medical school in Bali, a province in Indonesia. This private medical school was established in 2009. The clinical clerkship program began for the first time in 2013 at the main teaching hospital and in 2017 at the satellite teaching hospital. Since then, clinicians at both teaching hospitals who mainly provide healthcare services to patients have also been required to teach medical students as part of their daily duties.

### Data analysis

Qualitative data from the FGs and in-depth interviews were analysed using the thematic analysis method. Steps for Coding and Theorization (SCAT) was used to determine the themes and subthemes based on the verbatim transcripts. A structured matrix was used in SCAT to guide the analysis of the qualitative data.^28^ The development of themes was based on the 4S framework, which consists of situation, self, support and strategies.^22^^,^^24^ Two authors (SS and DS) independently reviewed each transcript after each FG or interview and identified keywords that were subsequently categorised into themes and subthemes. Any disagreements were resolved among all authors.

The analysis revealed four themes and 12 subthemes related to the 4S framework. Eight subthemes were identified from the first focus group, and one new subtheme emerged in the second focus group. New subthemes continued to be identified up to and including the ninth interview for a total of 12 subthemes overall. The analysis of the remaining interview transcripts did not result in any subthemes, which suggests that data saturation was reached. We carried out a final check with the members of the focus groups by providing the final summary to the study participants for review to ensure that the themes and subthemes were in accordance with the participants’ intents.

## Results

**Figure 1:**
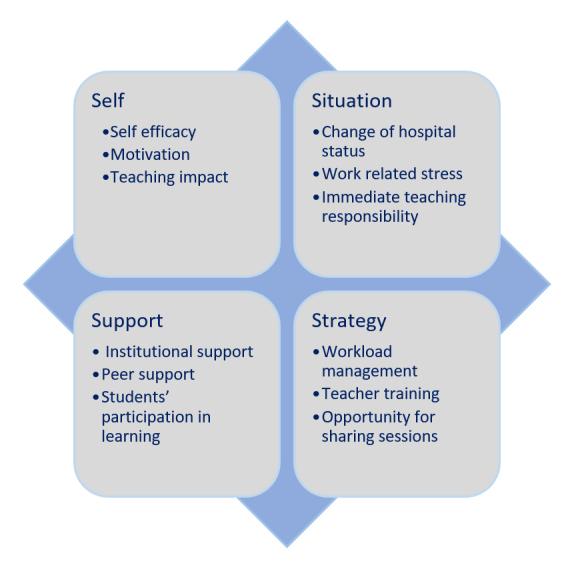
Identified themes and subthemes based on the 4S framework

Twelve subthemes across the four themes based on the 4S framework (situation, self, support, and strategies) were identified. Figure 1 was created to provide a comprehensive overview of all themes and subthemes identified during the study. We describe each theme and its corresponding subthemes below.

## Self

Several self factors influenced the participants’ identity as clinical teachers, including self-efficacy, motivation, and teaching impact. The participants further elaborated that their self-efficacy was affected by their perceptions of teaching, their evaluation of their teaching ability, how other people viewed their teaching role, and their prior experience in teaching.

Some participants stated that becoming a clinical teacher was a prestigious step in their careers. It made them proud and confident, and their role as teachers gave them a sense of added value.

“It is a feeling of pride, for sure. I never imagined a world like this. Now that I have to teach and share my knowledge, it is like I’ve been awarded the trust to teach the students. It makes me confident, proud of myself, and also creates a feeling of pride when I talk about it to the people around me. I feel like I have additional value as a clinical teacher, as if I have become a more trustworthy individual.” (FG13, F, <3 years, NC, R)

Initially, some participants questioned their ability to teach. Even after performing the role for a while, some participants still considered their performance suboptimal; however, other participants acknowledged that they did not know how to evaluate their own ability properly.

“Perhaps, as a clinician, I already provide optimal healthcare, but as a clinical teacher, I feel I’m still lacking in some way. What I mean is that I do not yet provide an optimal service to the students. First, because my understanding of what it means to be a clinical teacher is still lacking. Second, my service to patients is my primary obligation; therefore, my time-management skills still need improvement” (I12, M, >3 years, NC, I)

“One of the challenges we face is that we don’t know exactly how we should teach the students and create lessons that are attractive to them. I’m not able to assess myself…Perhaps the students think, ‘Ah, that is not interesting.’ So, they become passive [in the learning session]. We don’t [yet] know how to assess ourselves.” (FG8, M, >3 years, NC, I)

Most of the participants stated that their experience in teaching while undergoing their residency training boosted their self-efficacy as clinical teachers.

“During residency training, it was an obligation [for us] to guide junior clinicians in their residency program as well as [provide educational support to] undergraduate students. So, when this hospital becomes a teaching hospital, I am not worried about the need to teach as we have already been involved in teaching since the beginning of our residency training.” (FG7, M, <3 years, NC, Og)

A positive view from the general public regarding clinical teachers has also enhanced participants’ self-efficacy in teaching.

“Sure, other people’s [society’s] positive perspective of clinical teachers really had a positive impact on me. Although there were some barriers, such as duties involving patient care or the need for time management, I become more passionate [about teaching] because of the positive view from others.” (I12, M, >3 years, NC, I)

The participants’ motivation also influenced them when taking on the role of clinical teacher. Both internal and external motivations were identified in this study. For internal motivation, the participants considered teaching a personal call and a passion, whereas the external motivation identified by participants was from family members and students. Eventually, these external motivations are internalised, and the new clinical teacher becomes more eager to teach.

“Personally, I think [I am more motivated as a teacher] simply because my child is also a university student. To be honest, the way I teach my students is the same way I would like my child’s teachers to teach him. That is one of the reasons I am interested in becoming a teacher.” (FG2, M, <3 years, C, S)

Another feature related to the self factor was the participants’ perceptions of the impact of teaching. Most of the participants experienced positive impacts in becoming a clinical teacher, including a motivation to learn more and to improve patient care.

“I think that a clinician and a clinical teacher can complement each other. The role of clinical teacher supports our role as a clinician because [in both cases] we have to learn [new things]. Therefore, it’s possible that if a clinician is not a clinical teacher, he or she could lack the motivation to keep up-to-date on medical information. A clinician is skilled in good communication since it’s a daily activity to discuss [medical care] with patients and the patient’s family. Therefore, being a clinical teacher might come with an advantage since communication plays an important role in teaching. To conclude, both professions are supportive of each other.” (I8, M, >3 years, C, S)

“For me, there have been many changes in the way we care for and provide services to our patients. Before we became teachers, we usually provided one-sided services. Now, after becoming teachers, [we’ve] so many changes in patient care. Things are developing rapidly. So, [nearly every day] I learn something new.” (FG4, M, <3 years, C, I)

## Situation

Almost all participants said they became clinical teachers due to a change in the hospital’s status as a teaching hospital. Due to this change, there were participants who perceived that teaching was difficult in the beginning and that being a clinical teacher was little more than an obligation.

“One reason I chose to work in a regional hospital is because I thought I would just be involved in patient care. Then, when the hospital became a teaching hospital, I felt it was a burden in the beginning, as I believed I couldn’t teach others. As time passed and the hospital became a teaching hospital, like it or not, we were obliged to become clinical teachers.” (FG5, M, < 3 years, C, S)

Work-related stress also influenced the participants’ identity as clinical teachers. This work-related stress was mainly related to the limited time available due to patient care and teaching activities.

“To be honest, it was very difficult to manage my time between patient care and student learning. Currently, there are so many learning activities. Some of those activities could be done simultaneously while we provided patient care, such as bedside teaching; however, for some activities, like clinical tutorials, I’ve had to allocate special time for them.” (I1, F, <3 years, NC, Op)

There were also participants who considered teaching a burden because they were asked to immediately take on the responsibilities of teaching. One example concerns a participant who was immediately co-opted as a clinical teacher right after graduating from residency training.

“Perhaps because we’d just finished residency training, I wanted to stay away from the educational arena. We should have been given time to recover [from residency]. I personally think of it like that because, to me, teaching is burdensome. Thus, I try to enjoy it by sharing with others how to be a clinical teacher. I try to enjoy, even though it is an obligation.” (FG16, F, <3 years, NC, Ot)

## Support

Most of the participants agreed that institutional support in the form of training to understand the role of a clinical teacher was already sufficient; however, there was an expectation that the institution would facilitate them in their career development. In terms of the rewards of teaching, most participants discussed the non-material rewards they have received thus far.

“I think the institution’s recognition of us in the satellite teaching hospital is still lacking. Therefore, we don’t know how to actually use the system for our career development. We were told there were opportunities to develop ourselves, either in continuing education or otherwise. But it seems to be just talk – not more than that.” (FG2, M,<3 years, C, S)

“Based on [my] personal opinion, if a patient knows that I am teaching in a medical faculty, his/her level of trust will increase. As a result, any explanations that are given to the patient may be more readily accepted. That’s the benefit I’ve received as a clinical teacher.” (I5, F, >3 years, NC, D)

Peer support was also identified as a key support factor that influenced clinical teachers’ development of a professional identity. One form of peer support included helping each other to fulfil teaching duties.

“My colleagues and I in the department really support each other. We give advice to each other, share knowledge, and help to fulfil each other’s duties. For example, if someone is free at the hospital [and I am not], he or she can take over my teaching duties.” (I12, M, >3 years, NC, I)

Students’ participation in learning was also identified as a support factor for participants in enacting their teaching roles. Students’ active participation has made clinical teachers more excited to teach.

“In the outpatient clinic, students were instructed to examine a new patient; however, sometimes, no actions were taken by the students. This situation could make either the tutor or the lecturer feels bored or uninterested since only information was relayed, and no active discussions took place. Such a situation makes teaching boring. It’s more interesting when students actively ask us questions.” (FG7, M, <3 years, NC, Og)

## Strategies

Some participants shared how they managed their workload using both time-management strategies and coordination within the team. With regards to time management, each participant had his or her own way of managing their time, based on individual needs. As a result, both patient care and teaching became manageable. Coordination within the team included the distribution of work or assignments among colleagues.

“We receive patients from all outpatient clinics and wards. Most of the time, our working hours are fixed between 8:00 am and 1:00 pm. One of the challenges we encounter every day is the preparation of the compulsory morning briefing report. As a result, this creates a habit to prepare early. Before delivering patient care by 7:00 am, we would teach students the theory and explain the application afterwards.” (FG13, F, <3 years, NC, R)

“In this department, there are four clinical teachers. I’ve already assigned each clinical teacher to teach certain cases based on their competency level. So, according to their competency level, I know which cases we have to teach to the students and which cases can be supplementary.” (FG1, F, <3 years, C, R)

Most participants acknowledged that the clinical teacher training provided by the institution was one of the resources they relied on to improve their teaching skills.

“In my personal opinion, clinical education [for teachers] could be more challenging. Before joining this medical school, I was also a lecturer at another university. While there, I did not receive any clear directions about teaching. Now, however, more direction and guidance is provided through the institution’s training program.” (I11, F, <3 years, NC, D)

The participants stated that the opportunities to share experiences and ideas with other colleagues and peers were also a chance to hone their performance as clinical teachers.

“I first received information about being a clinical teacher from colleagues at this medical school. Sometimes, I also discuss things [about teaching] with friends in the same department at Hospital A. When we get together, we’ll often discuss how to teach students. If there’s a strategy I can apply in our current situation, I’ll do so because it isn’t possible to fully adopt the system at Hospital A, which has more resources.” (I9, M,>3 years, C, A)

## Discussion

Self-efficacy plays an important role in the transition period from clinician to clinical teacher because little is more pervasive than an individual’s belief in their ability to control their own motivations and behaviour.^22^ At the beginning of their career as clinical teachers, some participants were found to have low self-efficacy, as demonstrated by their feelings of low self-esteem as a teacher. It was also found that some participants were still having difficulties assessing themselves. This could negatively impact self-efficacy because, without self-assessment, it is difficult for clinical teachers to know what they’re doing well and what still needs improvement.^29^ Low self-efficacy could make clinical teachers feel that teaching is a burden, which can result in stress and depression and can narrow the teacher’s approach to problem-solving.^30^ Thus, a negative assessment of one’s teaching ability could lead to impaired development of professional identity.

One means of facilitating self-assessment ability in clinical teachers is through the use of the reflective practice as a clinical teacher. A study by Bajwa and colleagues compared two different formats for faculty development programs: personalised coaching or guided self-reflection.^31^ The study showed that both formats improved self-efficacy and the skills of junior clinical teachers; however, the personalised coaching method had several advantages. One advantage is that it supports the development of teaching communities by offering a safe environment for the participants to shape their professional identity as a teacher and strengthen a sense of belonging.^31^ Coaching can help individuals engage in reflective practice to grow and develop. Because coaching values and nurtures continuous self-reflection, a coach can encourage individuals to perform self-monitoring. This is an important aspect in helping clinical teachers develop an identity because identity formation aims to encourage individuals to reflect on their development as they adopt a specific role, either in their way of thinking or acting or feeling.^32^^,^^33^

Most participants assessed their changed role from clinician to clinical teacher as a result of an unanticipated change in their hospital’s status to that of a teaching hospital. Such an unanticipated change may be challenging because clinicians may not have the opportunity to prepare themselves.^22^ Therefore, institutions should implement a strategy that minimises the negative impact of unanticipated change. A peer mentor program could help clinical teachers navigate their new roles and internalise external motivation. This program could be led by appointed clinical teachers who have already successfully internalised their teaching roles to serve as peer mentors. Feedback from a peer mentor would assist in professional development and self-reflection.^34^ A study by Mylona and colleagues. showed that formal mentoring between clinical teachers has a positive impact on professional commitment and job satisfaction.^35^

Despite the change in hospital status that resulted in clinicians becoming clinical teachers, most participants described the positive impact of teaching. These positive experiences should be emphasised in faculty development programs to help clinical teachers recognise the connection between the roles of a clinical teacher and a clinician;^36^ however, some participants in this study felt that serving as a clinical teacher was a burden because the role started immediately after graduating from residency training. Unsolved stress or burnout during residency training could be an influential factor because stress and burnout can negatively impact an individual’s assessment of the teaching role. While immediate participation as a clinical teacher cannot always be avoided, measures, such as mentoring programs in which a mentor provides feedback and promotes the mentee’s self-reflection could be implemented to help clinical teachers reduce stress or burnout.^37^^,^^38^

Lankveld and colleagues recommended that workplaces recognise and reward teaching opportunities as a basis for career promotion.^36^ Some participants stated that the institution had expressed a commitment to providing appropriate opportunities to continue their education, but they felt that there was little progress. One important factor related to individuals’ perceptions of existing support is the intention of the support provider. If good intentions are not followed by real changes, it may lead to feelings of apathy in recipients of support when it eventually materializes.^39^

It was found that participants used direct coping strategies, including coordination in teams, to manage workloads and to share with peers to understand their role as clinical teachers. Studies have shown that the adoption of direct coping strategies is positively correlated with self-efficacy and work retention.^40^ It is expected that good self-efficacy and retention in carrying out the clinical teacher role will have a positive effect on the development of the professional identity of clinical teachers.

Peer discussion was also identified as a means of developing a teaching community of practice within an institution. Community of practice is aimed at encouraging a sense of belonging among participants by fostering a feeling of acceptance by others in a particular group. A sense of belonging and sharing practices through a community of practice are considered important factors in the development of professional identity.^41^ Through sharing practices, a community of practice can provide a collaborative network in which the people within the group maintain good personal relationships, enrich the perceptions toward teaching, develop shared understanding, and expand their knowledge.^41^^,^^42^

One strategy to develop a community of practice is to include peer reflection activities as part of a clinical teacher’s evaluation. A dialogue can be initiated between clinical teachers that eventually affects the reflection process of each clinical teacher. Based on the practice of self-reflection, clinical teachers will continue to construct and reconstruct a professional identity because professional identity develops continuously and is influenced by interactions with other parties. This dialogue process will also enhance collegiality and a sense of belonging and will provide emotional support through empowerment.^41^^,^^43^ The study by Boerboom and colleagues showed that peer reflection helps clinical teachers translate their students’ feedback into concrete plans related to their teaching roles and enhances their ability to reflect on their development critically.^44^

We have identified the importance of reflective ability in professional identity development. The implementation of a teaching portfolio can be used to promote reflective practice that can help clinical teachers articulate their personal conception of teaching. The personal conception may evolve through reflection, which could ultimately assist clinical teachers in enhancing their expertise in teaching and developing their professional identity.^45^^-^^48^

The study findings have implications for medical education, specifically faculty development programs for clinical teachers. Each of the 4S framework factors can be used to design a faculty development program relevant to the needs of clinical teachers. For example, the current study has further emphasised that self-reflection (self factor) and feedback from others (e.g., peer feedback, support factor) form a powerful combination in supporting transitions and identity formation.^47^ The role of the community of practice (strategy factor) as social support in understanding teaching roles and developing a professional identity as a clinical teacher is also highlighted. Thus, either formally or informally, we propose that interactions among members of a community of practice should be encouraged and nurtured. Formally, the medical education institution could intentionally create a community of practice as part of its faculty development program, such as by including peer feedback related to the teaching role, which is an important source for a teaching portfolio. Peer feedback can be used by clinical teachers to make decisions regarding their actions for improvement in the quality of teaching roles.^45^^,^^49^ Informally, the medical education institution might provide an online platform or a physical space for clinical teachers to share their experiences in teaching. As Lankveld and colleagues (2016) found in their study, informal teacher communities not only support professional development but also validate and strengthen the participants’ identities as teachers.^43^

This study is a qualitative study with the aim of exploring the process of professional identity development in clinical teachers in a relatively new medical school. It was conducted at a single medical school in Indonesia; thus, the study findings could be specific to this context. Furthermore, the exploration was cross-sectional; therefore, it might not be able to describe the process of developing professional identity over time. Nevertheless, the factors influencing clinical teachers’ professional identity, meaning those who were still undergoing a transition period from clinician to clinical teacher in a recently established teaching hospital, have been identified.

Further studies could explore whether the factors influencing a clinical teacher’s development of professional identity in other settings (e.g., across cultures or institutional status) would reveal similarities or differences. It would also be worthwhile to carry out a longitudinal study to explore these factors. In addition, studies designed to explore the use of a teaching portfolio as a reflective practice tool and the role of a community of practice would provide important insights further to understand the formation of clinical teachers’ professional identity.

## Conclusions

The 4S framework used in this study was found to be a useful paradigm in providing a comprehensive explanation of the factors influencing the development of professional identity in clinicians who are transitioning into clinical teachers as well as the importance of creating a needs-based faculty development program. Our study has revealed the importance of incorporating a community of practice into formal faculty development programs as well as equipping teachers with peer feedback processes and reflective practice skills, all of which can be honed through the development of a teaching portfolio. The factors identified in this study originated from the perspectives of clinical teachers within an Eastern educational culture and a relatively new medical school, which we believe will add to the breadth of literature on faculty development and the formation of professional identity in clinical teachers.

### Acknowledgement

The authors would like to thank Universitas Indonesia for funding this research through PUTI Grant with contract number NKB-2288/UN2.RST/HKP.05.00/2020.

### Conflict of Interest

The authors declare that they have no conflict of interest.
